# Supplementary material for: Loneliness among Homeless Individuals during the First Wave of the COVID-19 Pandemic
Source: Int J Environ Res Public Health. 2021 Mar 16;18(6):3035. doi: 10.3390/ijerph18063035 (PMC7999173; doi:10.3390/ijerph18063035)
Supplement: Supplementary file 1 [file ijerph-18-03035-s001.pdf]

Table S1. Results of sensitivity analysis, using full information likelihood approach.

Independent Variables	Coeff	SE	z	P>z	95% CI	
**Gender: Female (Ref.: male)**	-0.58	0.43	-1.36	0.18	-1.42	0.26
Age	-0.01	0.02	-0.49	0.62	-0.03	0.02
Marital Status (Ref.: married, living seperated from spouse):						
Single	1.60*	0.67	2.37	0.02	0.28	2.92
Divorced	1.17	0.91	1.28	0.20	-0.62	2.96
Widowed	1.30	0.75	1.72	0.09	-0.18	2.78
Level of Education (CASMIN Classification) (Ref: Primary):						
Secondary	-1.15	0.61	-1.87	0.06	-2.35	0.053
Tertiary	-0.24	0.94	-0.26	0.80	-2.08	1.60
Country of origin (Ref.: Germany):						
Neighbouring country	-0.56	0.48	-1.18	0.24	-1.50	0.38
Other EU- and non-EU countries	-0.33	0.43	-0.78	0.44	-1.17	0.51
Duration of homelessness (months)	0.00	0.00	0.98	0.33	-0.00	0.00
Share a sleeping space with more than three Persons: (from 1 = never to 4 = always)	0.36*	0.14	2.55	0.01	0.08	0.64
Chronic alcohol consumption (CDT >2.5%): Presence (Ref.: absence)	-0.14	0.41	-0.35	0.72	-0.94	0.65
Self-perceived risk of contracting COVID-19 (from 1 = very low to 5 = high)	0.39*	0.16	2.37	0.02	0.07	0.71
Constant	4.45	1.17	3.80	0.00	2.16	6.74

Unstandardized regression coefficients are displayed; robust standard errors in parentheses: * p<0.05.

Table S2. Determinants of loneliness among homeless individuals during the COVID-19 pandemic: Findings of multiple linear regressions, reference group marital status: divorced.

Independent Variables	Coeff.	SD	T	P> t	95% CI	
**Gender: Female (Ref.: male)**	-1.07*	0.42	-2.56	0.01	-1.91	-0.28
Age	0.00	0.02	0.11	0.91	-0.03	0.03
Marital Status (Ref.: divorced)):						
Married, living separated from spouse	-1.05	0.58	-1.82	0.07	-2.20	0.10
Widowed	-0.17	1.15	-0.15	0.88	-2.45	2.11
Single	0.28	0.56	0.50	0.62	-0.83	1.39
Level of Education (CASMIN Classification) (Ref.: Primary):						
Secondary	-0.99	0.61	-1.62	0.11	-2.20	0.22
Tertiary	0.39	0.96	0.41	0.68	-1.51	2.30
Country of origin (Ref.: Germany):						
Neighbouring country	-1.48**	0.51	-2.93	0.00	-2.49	-0.48
Other EU- and non-EU countries	-0.34	0.57	-0.60	0.55	-1.49	0.80
Duration of homelessness (months)	0.00	0.00	0.83	0.41	-0.00	0.01
Share a sleeping space with more than three Persons: (from 1 = never to 4 = always)	0.42*	0.17	2.45	0.02	0.08	0.79
Chronic alcohol consumption (CDT >2.5%): Presence (Ref.: absence)	0.18	0.53	0.34	0.74	-0.87	1.22
Self-perceived risk of contracting COVID-19 (from 1 = very low to 5 = high)	0.41*	0.18	2.30	0.02	0.06	0.77
Constant	4.53	1.09	4.15	0.00	2.36	6.70
Observations	94					
R^2	0.28					

Unstandardized regression coefficients are displayed; robust standard errors in parentheses * p<0.05.
